# Repurposing an ‘Old’ Drug for the Treatment of COVID-19-Related Cytokine Storm

**DOI:** 10.3390/jcm12103386

**Published:** 2023-05-10

**Authors:** Emanuele Pontali, Francesca Filauro

**Affiliations:** 1 Department of Infectious Diseases, Galliera Hospital, 16128 Genoa, Italy; 2 Pharmacy Service, Galliera Hospital, 16128 Genoa, Italy; francesca.filauro@galliera.it

## 1. Introduction

The severe acute respiratory syndrome coronavirus 2 (SARS-CoV-2) pandemic has hit more than 200 countries with more than 750 million confirmed cases and more than 6 million deaths worldwide [1]. The most severe forms are those manifesting with coronavirus disease-2019 (COVID-19)-related pneumonia and respiratory failure. These are the conditions where the highest mortality is observed. The role of the so-called cytokine storm has not been fully elucidated, but even if it is not always clinically evident in patients [2,3,4], there is clear evidence that pulmonary compartmentalization of hyperinflammation occurs [5,6,7,8].

In the early weeks of the expansion of the SARS-CoV-2 pandemic, several researchers tried to face the COVID-19-related cytokine storm. It was clear that what was happening in the lungs of patients with COVID-19 pneumonia was not only a direct viral effect, but also the result of an excessive immunological response [2,3,4,5,6]. Several different drugs acting on the interleukin (IL)-1–IL-6 pathway have been tested. Some of them (tocilizumab, baricitinib, anakinra, etc.) proved to have some efficacy [9].

## 2. Discussion

Anakinra is a recombinant IL-1 receptor antagonist that has been previously used in other hyperinflammatory situations, such as the macrophage activation syndrome complicating severe bacterial sepsis and the cytokine release syndrome observed during antitumoral chimeric antigen receptor (CAR) T-cell therapy [10,11,12]. At the start of the SARS-CoV-2 pandemic, it soon became clear that anakinra was a good candidate to be tested in the setting of COVID-19-related cytokine storm.

In the first months of 2020, several reports showed the efficacy of anakinra, but they included only small cohorts, and it was not fully clear in which patients anakinra showed its greatest activity [13,14,15,16,17,18,19,20]. Later, in 2021, a systematic review and patient-level meta-analysis by Kyriazopoulou et al. definitively showed that anakinra could be a safe, anti-inflammatory treatment option to reduce the mortality risk in patients admitted to hospital with moderate to severe COVID-19 pneumonia, especially in the presence of signs of hyperinflammation (C-reactive protein, CRP > 10 mg/dL) [21]. Later, a randomized controlled trial proved anakinra’s efficacy in reducing mortality in patients affected by COVID-19 when guided by levels of soluble urokinase plasminogen activator receptor (suPAR) [22].

Afterwards, several other papers reported additional experiences with anakinra in COVID-19 patients, comparisons with other immunomodulators such as tocilizumab and baricitinib, and attempts to clarify the exact role of this drug. Some of these papers were published in the *JCM* [9,23,24,25,26,27].

First, a Spanish study from the early phases of the epidemic (2020) studied different anti-cytokine therapies (tocilizumab, anakinra, baricitinib) in a specific fragile population, i.e., among kidney transplant recipients requiring hospitalization due to SARS-CoV-2 infection [23]. It was not possible to clarify the specific activity of anakinra because of the small size of the studied cohort (33 patients); nevertheless, 9 out of 33 patients received anakinra alone (6) or in combination with tocilizumab (3). The authors concluded that, notwithstanding the limits of the study, anti-cytokine treatment was safe among kidney transplant recipients, and responses, in terms of clinical efficacy, were favorable [23].

A large (10 intensive care units) French prospective cohort study conducted in 2020 and published in the *JCM* in 2021 evaluated the impact of tocilizumab and anakinra on critically ill COVID-19 patients [24]. Tocilizumab and anakinra were employed only in about 6-7% of the studied population. In particular, 24 patients (6.28% of the cohort) received anakinra. Different from many other studies, Ursino et al. concentrated their effort on determining the effect of anakinra and tocilizumab in a more severely affected population, i.e., intubated patients. They highlighted a significant effect of anti-cytokine treatment in increasing the chances of being successfully extubated [24].

In 2021, a new retrospective Spanish study compared the efficacy of anakinra and baricitinib, associated with corticosteroids, concerning the survival of patients hospitalized with COVID-19 pneumonia in two hospitals [25]. The main outcomes of this study were the need for invasive mechanical ventilation (IMV) and in-hospital death. The study included 125 and 217 individuals in the anakinra and baricitinib groups, respectively. Although comparable, the two groups differed in the use of remdesivir and the frequency of use of a second immunomodulatory drug (greater in the baricitinib group in both cases). In the end, after comparing outcomes and the cohorts’ characteristics, the authors concluded that the efficacy of the two drugs in preventing IMV and in-hospital death did not significantly differ [25].

In 2021, the *JCM* hosted a systematic review and meta-analysis regarding the impact of anakinra on the outcomes of hospitalized patients with COVID-19 carried out by a Greek group [26]. They included 6 studies reporting data on 1553 COVID-19 patients with moderate to severe pneumonia, where about 50% had been treated with anakinra. The results showed a pooled hazard ratio for death in patients treated with anakinra of 0.47 (95% confidence intervals 0.34 and 0.65). The authors concluded that their meta-analysis showed about a 50% decrease in the adjusted risk of death in hospitalized patients with moderate to severe COVID-19 treated with anakinra compared with patients that did not receive anakinra [26].

In 2022, in the *JCM*, a group from Romania reported their experience in the use of tocilizumab and anakinra, both in association with remdesivir, in patients with moderate to severe forms of COVID-19 pneumonia who had developed a cytokine storm (worsening of symptoms on days 8 to 10 of the disease, fever, dyspnoea with tachypnoea, decreased oxygen saturation, and increases in three parameters, namely CRP, LDH, and ferritin) [27]. In this study, 195 patients received remdesivir and anakinra. Such a combination treatment had a favorable clinical (improvement of clinical conditions, improved oxygen saturation) and laboratory (CRP, LDH, and ferritin approached normal values on the 10th day of treatment) effect, both in the moderate and severe forms [27].

The last paper of this series published in the *JCM* is a review of the evidence from pivotal trials that led to the approval of effective therapeutics in the treatment and prevention of COVID-19 [9]. Unfortunately, different from observational studies, randomized clinical trials returned discordant results on the efficacy of anakinra [21,28,29], probably because of the study design and population, choice of outcomes, timing, etc. In the end, the authors concluded that there is limited evidence of the place and role of anakinra in the treatment of COVID-19 [9].

## 3. Conclusions

In conclusion, there is increasing evidence of the positive role of anakinra in the treatment of moderate to severe COVID-19. Its most effective activity is likely to be observed in the early stages of COVID-19 and when there is hyperinflammation, measured as elevated CRP (>10 mg/dL) or suPAR (≥6 ng mL). More studies, even cohort and retrospective ones, will be useful in further elucidating anakinra’s role in COVID-19, and will be welcomed in the international literature.
